# Non-invasive respiratory support in SARS-CoV-2 related acute respiratory distress syndrome: when is it most appropriate to start treatment?

**DOI:** 10.1186/s12931-022-02258-5

**Published:** 2022-12-03

**Authors:** Riccardo Nevola, Antonio Russo, Samuel Scuotto, Simona Imbriani, Concetta Aprea, Marianna Abitabile, Domenico Beccia, Chiara Brin, Caterina Carusone, Francesca Cinone, Giovanna Cirigliano, Sara Colantuoni, Domenico Cozzolino, Giovanna Cuomo, Micol Del Core, Klodian Gjeloshi, Aldo Marrone, Giulia Medicamento, Luciana Agnese Meo, Francesco Nappo, Andrea Padula, Pia Clara Pafundi, Roberta Ranieri, Carmen Ricozzi, Luca Rinaldi, Ciro Pasquale Romano, Rachele Ruocco, Carolina Ruosi, Annabella Salvati, Ferdinando Carlo Sasso, Ausilia Sellitto, Pino Sommese, Angela Villani, Nicola Coppola, Luigi Elio Adinolfi

**Affiliations:** 1 Internal Medicine Unit, COVID Center, Department of Advanced Medical and Surgical Sciences, University of Campania “Luigi Vanvitelli”, 80100 Naples, Italy; 2 Infectious Diseases Unit, COVID Center, Department of Mental Health and Public Medicine, University of Campania “Luigi Vanvitelli”, Naples, Italy; 3 GEMELLI GENERATOR-Facility of Epidemiology and Biostatistics, Fondazione Policlinico Universitario Agostino Gemelli, Rome, Italy; 4 Internal Medicine and Hepatology Unit, Betania Evangelical Hospital, Naples, Italy

**Keywords:** SARS-CoV-2, COVID-19, ARDS, CPAP, NIV

## Abstract

**Background:**

Acute respiratory distress syndrome (ARDS) is one of the most severe complications of SARS-CoV-2 infection. Non-Invasive Respiratory Support (NRS) as Continuous Positive Airway Pressure (CPAP) and/or Non-Invasive Ventilation (NIV) has been proven as effective in the management of SARS-CoV-2-related ARDS. However, the most appropriate timing for start NRS is unknown.

**Methods:**

We conducted a prospective pilot study including all consecutive patients who developed moderate SARS-CoV-2-related ARDS during hospitalization. Patients were randomly divided into two intervention groups according to ARDS severity (assessed by PaO_2_/FiO_2_-P/F) at NRS beginning: group A started CPAP/NIV when P/F was ≤ 200 and group B started CPAP/NIV when P/F was ≤ 150. Eligible patients who did not give their consent to CPAP/NIV until the severe stage of ARDS and started non-invasive treatment when P/F ≤ 100 (group C) was added. The considered outcomes were in-hospital mortality, oro-tracheal intubation (OTI) and days of hospitalization.

**Results:**

Among 146 eligible patients, 29 underwent CPAP/NIV when P/F was ≤ 200 (Group A), 68 when P/F was ≤ 150 (Group B) and 31 patients agreed to non-invasive treatment only when P/F was ≤ 100 (Group C). Starting NRS at P/F level between 151 and 200 did not results in significant differences in the outcomes as compared to treatment starting with P/F ranging 101–150. Conversely, patients undergone CPAP/NIV in a moderate stage (P/F 101–200) had a significantly lower in-hospital mortality rate (13.4 vs. 29.0%, p = 0.044) and hospitalization length (14 vs. 15 days, p = 0.038) than those in the severe stage (P/F ≤ 100). Age and need for continuous ventilation were independent predictors of CPAP/NIV failure.

**Conclusions:**

Starting CPAP/NIV in patients with SARS-CoV-2-related ARDS in moderate stage (100 > P/F ≤ 200) is associated to a reduction of both in-hospital mortality and hospitalization length compared to the severe stage (P/F ≤ 100). Starting CPAP/NIV with a P/F > 150 does not appear to be of clinical utility.

## Background

Acute hypoxemic respiratory failure (hARF) is one of the most serious complications of SARS-CoV-2 infection, evolving into acute respiratory distress syndrome (ARDS). Its optimal management is still much debated. The efficacy of Non-Invasive Respiratory Support (NRS) as Continuous Positive Airway Pressure (CPAP) and/or Non-Invasive mechanical Ventilation (NIV) in SARS-CoV-2-related hARF is controversial [1–3], even if several studies support CPAP use [4–10]. A trial of CPAP seems to significantly reduce mortality and oro-tracheal intubation (OTI) rates compared to conventional oxygen therapy in patients with SARS-CoV-2-related ARDS [10]. Furthermore, a delay in the timing of OTI does not seem to worsen mortality and morbidity rates in critically ill patients with COVID-19 [9].

Application of a positive end-expiratory pressure (PEEP) through CPAP can prevent alveolar collapse and promote recruitment of already collapsed alveoli, thus improving ventilation of poorly ventilated though adequately perfused ones and reducing the shunt volume [2]. Moreover, compensatory mechanisms of hypoxic vasoconstriction (Euler-Lilijestrand mechanism) are inadequate in SARS-CoV-2-related ARDS, resulting in normal perfusion of poorly ventilated alveoli, with consequent severe hypoxemia [2]. The recruitment of these alveoli through PEEP would significantly improve hypoxia and may prevent OTI. In addition mortality rates in patients with COVID-19-related ARDS undergoing OTI and invasive mechanical ventilation (IMV) appear extraordinarily high [4, 9, 11–16], up to 97% during first pandemic waves, also due to the associated risk of bacterial superinfection [13]. More recently Peñuelas et al. [17] reports an overall 180-day survival rates of 59% in patients undergone IMV for COVID-19. During pandemic, the availability of intensive care units (ICUs) beds may also be poor. For all these reasons, an optimized and appropriate use of CPAP could represent a valuable weapon to the clinician.

CPAP has been reported to be effective in SARS-CoV-2-related hARF but, at present, the most appropriate timing to start treatment is unknown [3, 6, 18]. In this regard, Italian and English guidelines encourage new controlled studies on CPAP and NIV to define the role and timing for its use [18, 19]. Currently no standardized criteria for starting NRS have been defined. Clinical trials comparing the impact of CPAP/NIV on major outcomes when started at different severity levels of SARS-CoV-2-related ARDS (assessed by the PO_2_/FiO_2_-P/F ratio at blood gas analysis) as whether an early treatment can favor positive outcomes to date are not available.

The aim of this study was to evaluate if the timing of CPAP/NIV started at different P/F ratio levels could influence the outcomes (in-hospital mortality, OTI rate, hospitalization length) in patients with ARDS due to SARS-CoV-2 pneumonia.

## Materials and methods

### Study design

A prospective pilot study was carried out at the two Covid Centers of Policlinic Hospital, University of Campania "L. Vanvitelli", Naples. All patients with SARS-CoV-2 infection consecutively admitted to the Covid Centers of Internal Medicine and Infectious Diseases Units from December 13, 2020 to May 13, 2021, were evaluated. Diagnosis of ARDS and classification as mild (200 > PaO_2_/FiO_2_ ≤ 300), moderate (100 > PaO_2_/FiO_2_ ≤ 200) or severe (PaO_2_/FiO_2_ ≤ 100) were placed according to the Berlin Criteria [20].

Among all admitted patients with SARS-CoV-2 infection, only those who developed a PaO_2_/FiO_2_ (P/F) ratio ≤ 200 during hospitalization were enrolled in the study. Patients who already showed a P/F ≤ 200 at admission were excluded to avoid different standards of care prior to enrollment, which could potentially affect the results. Patients with hypercapnic respiratory failure and patients with contraindications to NRS, such as hemodynamic instability (systolic blood pressure < 90 mmHg despite fluid resuscitation), coma (Glasgow Coma Scale—GCS < 8) or non-compliant, were also excluded.

**Fig. 1:**
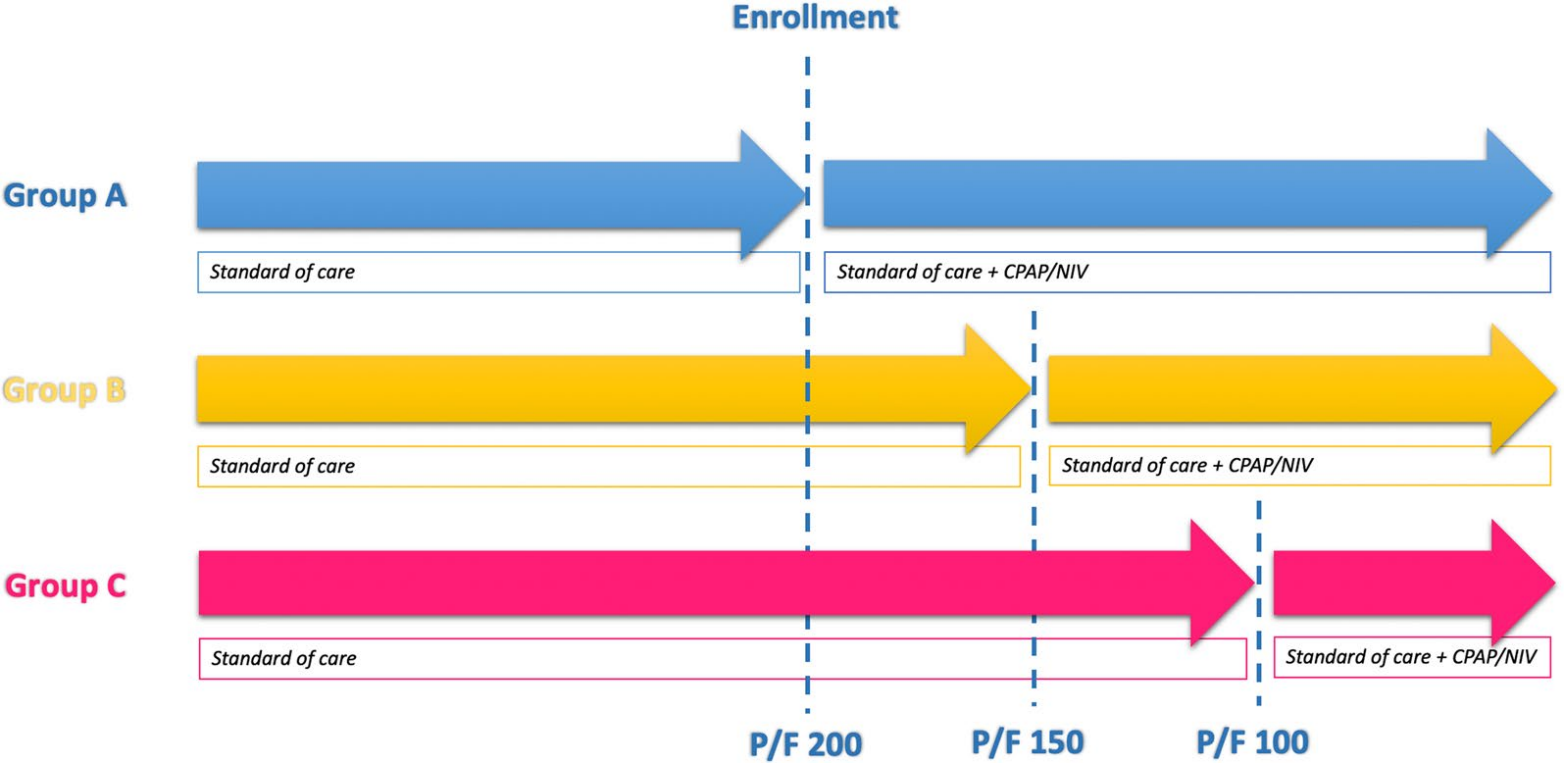
Study design: Group A started CPAP/NIV when P/F fell below 200, Group B underwent CPAP/NIV when P/F fell below 150. Group C derives from patients initially excluded from the study for refusal of non-invasive treatment and who subsequently initiated treatment with CPAP/NIV in a severe stage of ARDS (P/F ≤ 100). *CPAP* Continuous Positive Airway Pressure, *NIV* Non-Invasive Ventilation, *P/F* PaO_2_/FiO_2_ rate

Enrolled patients were assigned to two intervention groups in relation to the ward they were admitted to. Patients admitted to the Covid Center of Internal Medicine Unit (group-A) underwent CPAP/NIV as soon as they developed a P/F below 200. Conversely, patients admitted to the Covid Center of Infectious Disease Unit (group-B) were treated with CPAP/NIV when the P/F level fell below 150. Beyond NRS treatment, both groups were guaranteed the same standard of care (see below). The study design is shown in Fig. 1.

Part of the eligible patients (development of moderate respiratory distress during hospitalization) did not initially consent to CPAP/NIV treatment and were excluded from the study. A proportion of these patients gave consent to treatment in a severe phase of respiratory distress (P/F ≤ 100) and were also compared to those treated in moderate stage (groups-A and B, Fig. 1). The subgroup of patients who started the treatment in a severe stage of ARDS was identified as group-C.

### Data collection

Diagnosis of SARS-CoV-2 infection was done by RT-PCR test of nasopharyngeal swab sample [21]. On admission, all patients underwent anamnestic data collection, physical examination, blood tests and gas analysis, chest X-ray and lung ultrasound evaluation (standardized using the Lung Ultrasound score—LUS) [22]. A high-resolution chest tomography (HRCT) was performed. CT severity score was used for quantitative severity assessment by evaluating the extent of lung damage due to SARS-CoV-2 infection [23].

### Treatments

Medical treatments, respiratory support and clinical monitoring were performed in accordance with national and international guidelines.

NRS treatment was sequential. All patients who developed a P/F ≤ 200 (group-A) or ≤ 150 (group-B) during hospitalization started CPAP. Patients with inadequate response to CPAP were treated with NIV. In case of NIV failure, patients underwent OTI and IMV.

Initial PEEP was uniformly set at 7 cmH_2_0, with titration upwards or downwards according to clinical response (peripheral oxygen saturation—SpO_2_, respiratory rate—RR, blood gases) and patient's tolerance. The delivered oxygen (FiO_2_) was titrated to guarantee SpO_2_ > 92%. We used low starting pressures to reduce risk of pneumothorax and/or pneumomediastinum [24]. Patients who showed an acceptable response (SpO_2_ ≥ 92%) to High Flow Nasal Cannula (HFNC) were guaranteed oral feeding and CPAP support < 18 h/day. For patients who did not reach an acceptable SpO_2_ (< 92%) on HFNC, continuous CPAP (> 18 h/day) and total parenteral nutrition were provided.

Patients on CPAP without any clinical improvement (SpO_2_ and/or P/F) after a 4–6 h trial and/or developing signs of respiratory fatigue (RR > 30 acts/min, increased lactate, activation of accessory respiratory muscles) and/or hypercapnia were converted to NIV (modality: Pressure Support Ventilation, PSV) by applying a pressure support (PS). Initial PS were set at an intermediate level, starting from 6–8 cmH_2_O with titration upwards to ensure a TV of 7–8 ml/kg or downwards, if not tolerated [25].

Patients either with hypoxemia unresponsive to NIV (PaO_2_ < 60 mmHg) or with P/F persistently < 100 despite NRS (at least 6 h long) or who develop hemodynamic instability, underwent rapid OTI and IMV, in absence of contraindications or patient refusal (DNI).

Conversely, patients with a P/F ≥ 200 and a RR < 25 acts/min after 48 h of NRS weaned fast if they underwent only CPAP. Otherwise, PS was progressively reduced to initial levels and then converted to CPAP if they underwent NIV. Subsequently, oxygen was supplemented by HFNC or nasal cannulae, according to clinical needs.

The chosen interface for CPAP was the helmet, for NIV a full-face mask was used instead (Dimar, Medolla—Italy). CPAP/NIV was delivered by a compressed gas-based ventilator (ResMed Astral 150, San Diego, United States) connected to the interface through a bi-tube circuit. To reduce aerosolization, filters were interposed in the expiratory circuit. Healthcare personnel was equipped with complete body protection (double gloves, long-sleeved water-resistant gowns, goggles/face shields) and filtering face-piece (FFP)-3 masks.

Constant monitoring of patients' conditions and immediate OTI in case of NRS failure were guaranteed by a telemetry system (evaluation of vital parameters: SpO_2_, blood pressure, heart and respiratory rate, body temperature) and 24 h video-surveillance. Respiratory function was assessed with blood gas analysis, once daily or more frequently according to clinical status. In case of worsening, a chest imaging (ultrasound/X-ray/CT) was repeated.

Drug treatment included steroids (dexamethasone 6 mg/day for 10 days), low-molecular weight heparin (LMWH, prophylactic or therapeutic dosage depending on clinical needs), remdesivir (1st day 200 mg, 2nd-5th day 100 mg, only in patients within 10 days from symptoms’ onset) and tocilizumab (8 mg/kg once), according to the indications of National Institute of Health (NIH) and Italian Drugs Agency (AIFA) [26, 27]. Antibiotic therapy was given to all patients with evidence of bacterial superinfection.

### Outcome

The primary endpoints were in-hospital mortality. Secondary endpoints were: OTI rate and overall hospitalization length. The composite outcome of death and/or need for OTI was used to define CPAP/NIV failure in the overall population.

### Statistical analysis

Given the exploratory design of the study and no data available in literature on this topic to date, no formal sample size calculation is needed. However, due to the real-life nature of the study, we collected data on all consecutive patients with COVID-19 satisfying the inclusion criteria. All variables were summarized by descriptive statistics techniques. In depth, qualitative variables were presented as absolute and relative frequencies. Quantitative data, indeed, were summarized either as mean and standard deviation (SD), if normally distributed, or median and interquartile range (IQR), otherwise. Their distribution was previously assessed by the Shapiro–Wilk test. Between groups differences were analyzed either by Pearson Chi-square or Fisher exact test for qualitative variables, as appropriate. Student t-test or Mann–Whitney U-test were instead computed as for quantitative variables, according to their distribution. Potential predictive factors of the composite outcome of mortality and OTI were assessed by a univariate Cox regression model, with days between symptoms and death/OTI as time-for-event variables. Statistically significant variables at univariate analysis were included in a multivariable Cox regression model, consistently with the number of events. Overall survival and OTI rates were further summarized by Kaplan–Meier curves. A p-value < 0.05 was considered statistically significant. All analyses were performed by STATA 16 software (STATA Corp.).

## Results

**Fig. 2:**
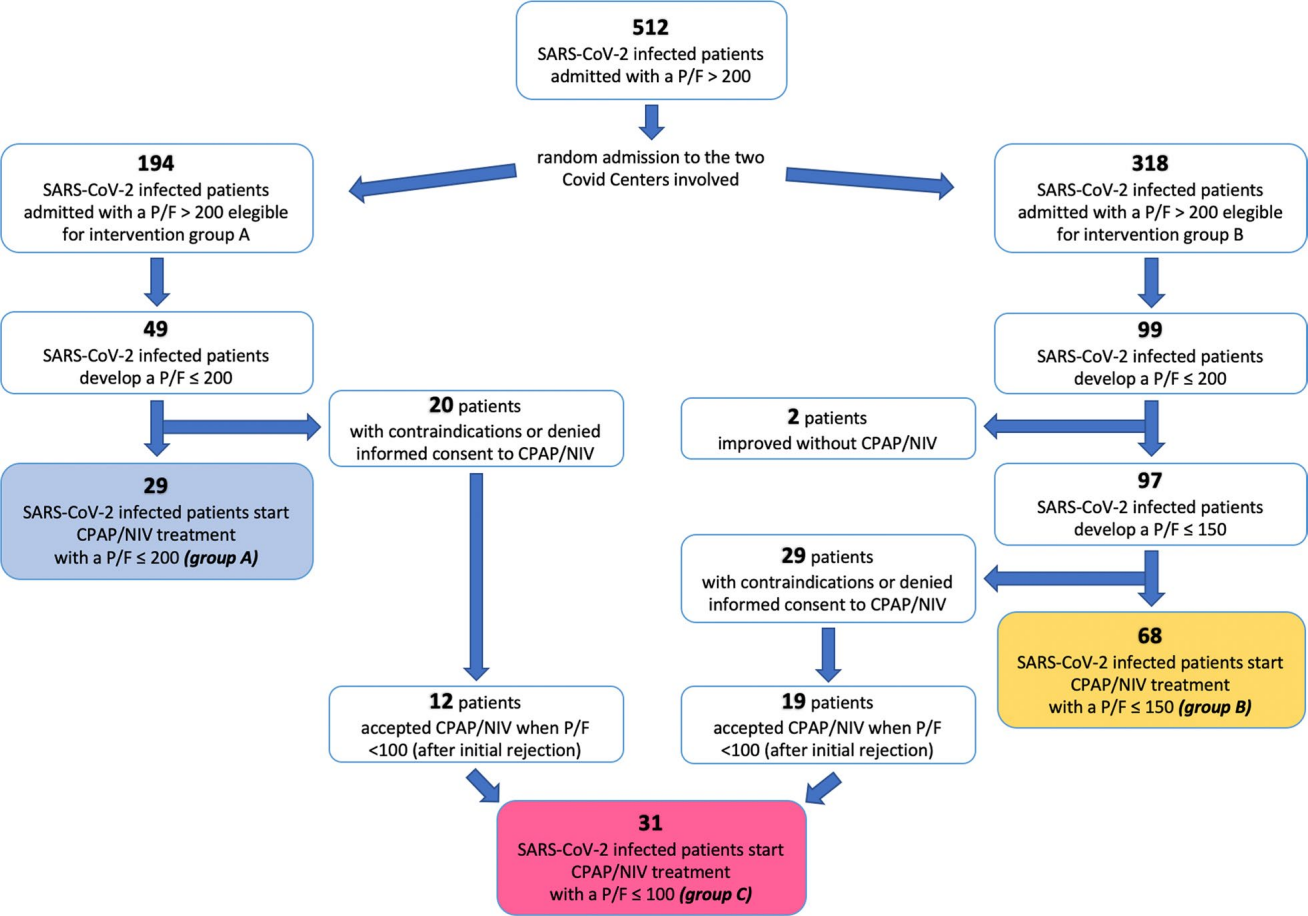
Flow chart of evaluated SARS-CoV-2 infected patients. *CPAP* Continuous Positive Airway Pressure, *NIV* Non-Invasive Ventilation, *P/F* PaO_2_/FiO_2_ rate

Of 512 SARS-CoV-2 infected patients admitted with a P/F > 200, 194 were admitted to the Covid Center of Internal Medicine Unit (group-A) and were eligible to start CPAP/NIV treatment if they developed a P/F ≤ 200; 318 patients were admitted to the Covid Center of Infectious Disease Unit (group-B) and were eligible to initiate CPAP/NIV if they developed a P/F ≤ 150 (Fig. 2).

In intervention group-A, of the 194 eligible patients admitted, 49 developed a P/F ≤ 200. Of these, 20 patients initially refused (14/20) or had ongoing contraindications (6/20: 3 septic shock, 2 hypercapnic respiratory failure, 1 coma) to NRS. Therefore, 29 patients were finally enrolled in the intervention group-A and underwent CPAP when P/F was ≤ 200 (Fig. 2).

In intervention group-B, of the 318 eligible patients admitted, 99 developed a P/F ≤ 200 and 97 of these (97/99, 98%) demonstrated further worsening to a P/F ≤ 150. Of these, 29 patients initially refused (22/29) or had ongoing contraindications (7/29: 2 septic shock, 3 hypercapnic respiratory failure, 2 coma) to NRS and 68 patients were finally enrolled in the intervention group-B and underwent CPAP when P/F was ≤ 150 (Fig. 2).

After initial refusal, 31 eligible patients consented to CPAP/NIV treatment when P/F was ≤ 100 and were subsequently enrolled as Group-C (Fig. 2).

The overall mortality rate among patients treated with CPAP/NIV was 17.2% (n = 22). According to the study methods, 33 cases of CPAP failure (25.8% of the total, subsequently treated with NIV) and 10 cases of NIV failure (30.3% of patients requiring PS, subsequently undergone OTI and IMV) have been reported. No infection among health personnel was recorded throughout the study period.

### Baseline characteristics and outcome of patients with moderate ARDS according to P/F above or below 150

Baseline characteristics and outcomes of populations with moderate ARDS and P/F between 151 and 200 (group-A) and between 101 and 150 (group-B) are summarize in Tables 1 and 2.

**Table 1:** Baseline characteristics of SARS-CoV-2 infected patients included in the study

Variables	All patients	150 < P/F ≤ 200 (A)	100 < P/F≤ 150 (B)	100 < P/F≤ 200 (A + B)	P/F ≤ 100 (C)	p		
						A vs. B	B vs. C	A + B vs. C
N	128	29	68	97	31			
Age, median [IQR], y	66,5 [58–73]	65 [54–72]	68 [59–72]	68 [59–72]	66 [58–72]	0.25	0.75	0.95
Sex: Male, n (%)	81 (63.3)	21 (72.4)	42 (61.8)	63 (64.9)	18 (58.1)	0.31	0.82	0.51
CCI, median [IQR]	3 [2–4]	2 [1–4]	3 [2–4]	3 [2–4]	3 [2–4]	0.141	0.28	0.64
Most relevant comorbidities								
COPD, n (%)	22 (17.2)	6 (20.7)	15 (22.1)	21 (21.6)	1 (3.2)	0.88	**0.02**	**0.025**
Diabetes, n (%)	32 (25.0)	6 (20.7)	17 (25.0)	23 (23.7)	9 (29.0)	0.65	0.60	0.49
Arterial hypertension, n (%)	78 (60.9)	15 (51.7)	47 (69.1)	62 (63.9)	16 (51.6)	0.10	0.13	0.3
Obesity, n (%)	23 (18.0)	4 (13.8)	10 (14.7)	14 (14.4)	9 (29.0)	0.91	0.13	0.06
Chronic kidney disease, n (%)	6 (4.7)	1 (3.4)	4 (5.9)	5 (5.1)	1 (3.2)	0.61	1	1
History of cancer, n (%)	4 (3.1)	1 (3.4)	**1** (1.5	2 (2.1)	2 (6.4)	0.51	0.23	0.25
Ischemic heart disease, n (%)	10 (7.8)	3 (10.3)	5 (7.3)	8 (8.2)	2 (6.4)	0.69	1	1
Congestive heart failure, n (%)	4 (3.1)	1 (3.4)	2 (2.9)	3 (3.1)	1 (3.2)	1	1	1
Smoking, n (%)	49 (38.3)	10 (34.5)	27 (39.7)	37 (38.1)	12 (38.7)	0.63	0.42	0.95
Duration before admission, median [IQR]	8 (5–10)	8 (3–11)	7 (5–10)	8 (5–10)	8 (5–10)	0.88	0.98	0.99
Arterial blood gases on admission								
PaO_2_, median [IQR], mmHg	66,6 (58.9–76.1)	68 (63.2–77.7)	68.5 (59.9–78.7)	68.05 (60.5–78)	58.6 (52.8–67.5)	0.94	0.82	0.80
PaCO_2_, median [IQR], mmHg	33.6 (30.8–38.2)	37 (33.5–39.5)	33 (30–37)	34.05 (31–38.1)	33 (29.8–37.1)	0.35	0.36	0.40
pH, median [IQR],	7.46 (7.44–7.5)	7.46 (7.45–7.49)	7.46 (7.44–7.51)	7.46 (7.44–7.5)	7.49 (7.47–7.5)	0.96	0.91	0.80
Lactates, median [IQR], mmol/L	1 (0–1)	0.8 (0.7–1.15)	1.4 (1.05–1.7)	1.2 (0.9–1.6)	1.9 (1.5–2.35)	0.60	0.63	0.79
Blood chemistry tests on admission								
WBC, cells/µL	8.04 (5.29–11.06)	5.76 (4.13–10.15)	8.69 (6.35–11.07)	8.21 (5.26–11.06)	9.33 (6.19–10.9)	0.07	0.71	0.27
Lymphocytes, cells/µL	800 (600–1120)	780 (560–1260)	800 (597–1032)	800 (575–1090)	750 (615–1124)	0.49	0.99	0.85
CRP, mg/dl	5.3 (2.58–12.8)	9.5 (3.66–13.67)	5.95 (2.38–11.9)	6.5 (2.5–13.25)	4.7 (2.58–8.96)	0.09	0.54	0.27
PCT, ng/ml	0.1 (0.04–0.27)	0.1 (0.04–0.17)	0.08 (0.03–0.33)	0.09 (0.03–0.24)	0.1 (0.06–0.5)	0.41	0.16	1
eGFR, mL/min/1.73m2	87 (70–97)	88 (73–97)	83 (71–98)	87.5 (71–98)	85 (69–92)	0.65	0.75	0.60
D-dimer, µg/L	555 (288–1075)	625 (267–977)	455 (301–955)	465 (285–1012)	815 (300–1420)	0.26	0.19	0.16
Na, mmol/L	137 (135–139)	136 (136–138)	137 (135–139)	137 (135–139)	137 (135–139)	0.91	0.87	0.87
Radiological imaging on admission								
LUS	16 (12–21)	18 (11–22)	16 (14–18)	16 (14–20)	15 (8–18)	0.70	0.29	0.19
CT severity score	12 (9–13)	11 (9–12)	12 (10–14)	12 (9–13)	11 (8–12)	0.35	0.29	0.41

Statistically significant values in bold

*CCI* Charlson Comorbidity Index, *COPD* Chronic Obstructive Pulmunary Disease, *CRP* C-Reactive Protein, *CT* Computed Tomografy, *eGFR* esitmated Glomerular Filtration Rate, *LUS* lung ultrasound score, *Na* sérum sodium, *Pa02* arterial partial pressure of oxygen, *PaCO2* arterial partial pressure of carbon dioxide, *PCT* Procalcitonin, *P/F* PaO2/FiO2 rate, *WBC* White Blood Cells

**Table 2:** Outcomes and characteristics of the treatments of group A (150 < P/F ≤ 200) vs. group B (100 < P/F ≤ 150) and group A + B (moderate ARDS, 100 < P/F ≤ 200) vs. group C (severe ARDS, P/F ≤ 100)

Outcomes and treatments	Group A (n = 29)	Group B (n = 68)	Group A + B (Moderate ARDS) (n = 97)	Group C (Severe ARDS) (n = 31)	p		
					A vs. B	B vs. C	A + B vs. C
Outcomes							
Mortality, n (%)	4 (13.8)	9 (13.2)	13 (13.4)	9 (29.0)	1	0.059	**0.044**
OTI, n (%)	2 (6.9)	8 (11.8)	10 (10.3)	4 (12.9)	0.72	1	0.69
Days of hospitalization, median [IQR]	13 [10–17]	15 [12–19]	14 [12–19]	15 [12–19]	0.25	0.08	**0.038**
CPAP: PEEP max, median [IQR], mmHg	7 [7–7.8]	8 [7–8.25]	7.5 [7.8]	7.5 [7.8]	0.12	0.41	0.25
PSV: PEEP max. median [IQR], mmHg	10 [6–10]	8 [5–8]	8 [5.25–8]	8 [6–8.25]	0.23	0.29	0.46
PSV: PS max, median [IQR], mmHg	5.5 [4.75–6]	6 [4–8]	6 [4–8]	5 [4.5–7.5]	0.59	0.79	0.87
Continuous ventilation (≥ 18 h/day), n (%)	7 (24.1)	15 (22.1)	22 (22.7)	17 (54.8)	0.82	**0.002**	**0.0007**
Overall days of ventilation, median [IQR]	5 [3–9]	6 [4–9]	6 [4–9]	6 [4–9]	0.94	0.79	0.86
Days from start NRS to OTI, median [IQR]	10.5 [NA]	7 [3–12]	7 [4–13]	4.5 [4–8.75]	0.50	0.648	0.41
Days from start NRS to weaning, median [IQR]	5 [3–11]	6 [4–9]	6 [4–9]	6.5 [4–9.25]	0.543	0.533	0.375
Conversion rate to NIV, n (%)	5 (17.2)	13 (19.1)	18 (18.6)	15 (48.4)	0.83	**0.002**	**0.003**
Tocilizumab, n (%)	3 (10.3)	10 (14.7)	13 (13.4)	4 (12.9)	0.75	1	0.94
Remdesivir, n (%)	12 (41.4)	20 (29.4)	32 (33.0)	3 (9.7)	0.25	**0.041**	**0.011**
Steroids, n (%)	29 (100)	68 (100)	97 (100)	31 (100)	1	1	1
Heparin, n (%)	29 (100)	68 (100)	97 (100)	31 (100)	1	1	1

Statistically significant values in bold

*CPAP* Continuous Positive Airway Pressure, *IQR* interquartile range, *NA* Not Applicable, *NRS* Non-Invasive Respiratory Support, *OTI* oro-tracheal intubation, *PEEP* Positive end-expiratory pressure, *P/F* PaO_2_/FiO_2_ rate, *PS* Pressure Support, *PSV* Pressure Support Ventilation

Age, Charlson Comorbidity Index (CCI), prevalence of each comorbidity, duration of symptoms before admission, blood tests, gas analysis and/or radiological parameters do not show any statistically significant difference at admission (Table 1). Likewise, there is also no significant difference in ventilation modalities (pressures, need for PS or continuous ventilation, ventilation days) or in medications used (Table 2).

The CPAP failure rate and the need to convert to NIV were 17.2% and 19.1% in groups-A and B, respectively. The NIV failure rate and the need for OTI and therefore IMV was 6.9% and 11.8% in groups-A and B, respectively. The overall mortality rate among patients treated with CPAP/NIV in moderate ARDS was 13.4% (n = 13).

The initiation of NRS at a P/F level between 151 and 200 does not result in a statistically significant difference for in-hospital mortality rate (13.8% group-A, 13.2% group-B, p = 1), OTI rate (6.9% group-A, 11.8% group-B, p = 0.72) and hospitalization length (p = 0.25) as compared to NRS started at a P/F level between 101 and 150 (Table 2). Furthermore, no statistically significant difference emerged between the two groups in pressure (PEEP/PS) used, need for PS or continuous ventilation, nor for ventilation length and days between the start of NRS and OTI or weaning.

**Fig. 3:**
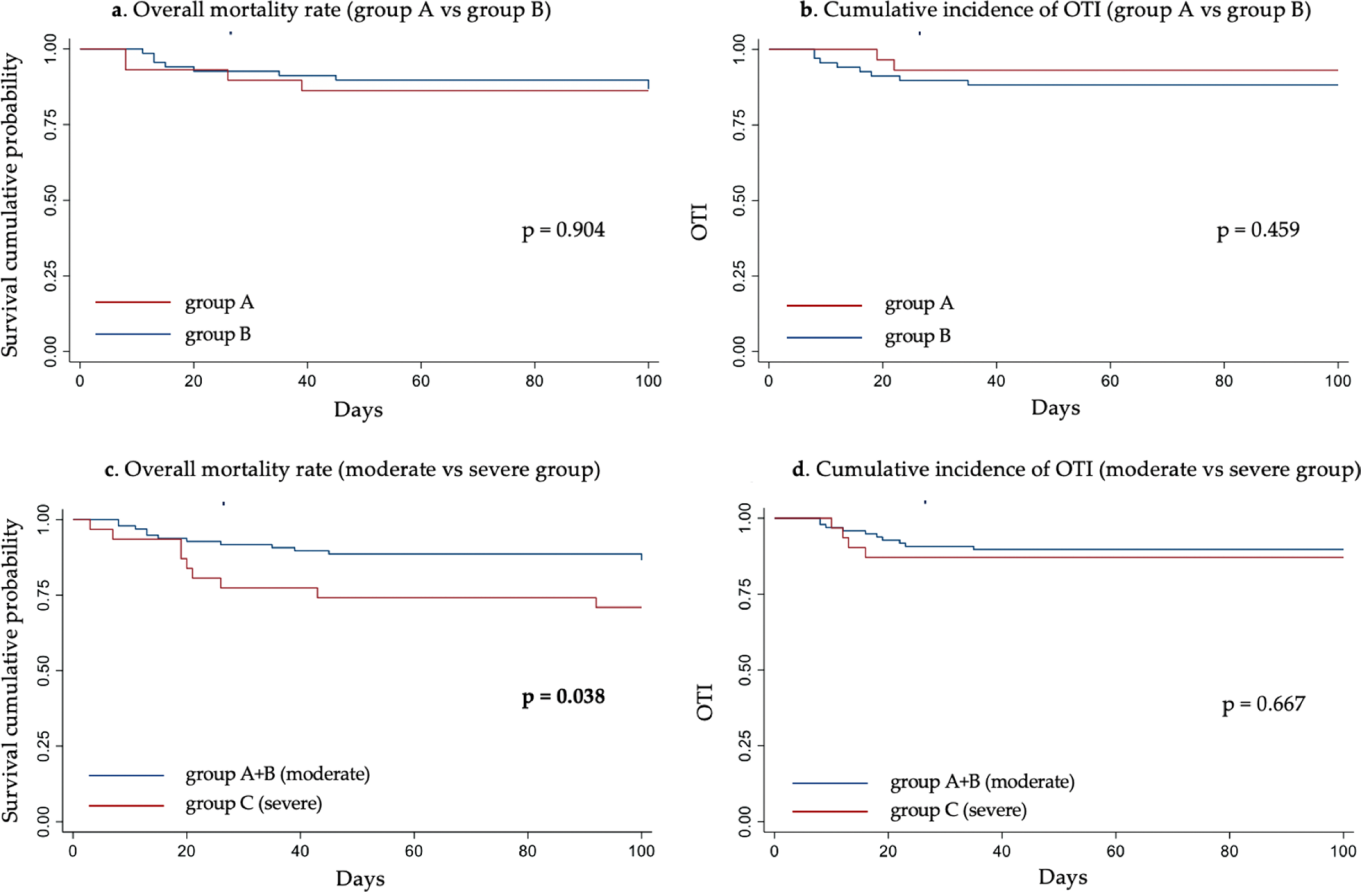
*Above:* Kaplan–Meier curves related to the analysis of mortality rates (**a**) and overall oro-tracheal intubation (OTI, **b**) among patients undergoing treatment with CPAP/NIV with a P/F between 151 and 200 (group A) and patients who initiated the treatment with a P/F between 101 and 150 (group B). *Below:* Kaplan–Meier curves related to the analysis of mortality (**c**) and OTI (**d**) rates among patients undergone CPAP/NIV in a moderate degree of respiratory distress (group A + B, P/F 101–200) and patients referred for treatment in a severe degree of respiratory distress (group C, P/F ≤ 100)

Figure 3a, b further show no significant differences in overall survival and OTI rates between patients undergoing NRS at P/F level 151–200 and 101–150.

### Baseline characteristics and outcomes of group-B (100 < P/F ≤ 150) vs. group-C (P/F ≤ 100) and patients with moderate (group A + B) versus severe (group-C) ARDS

An analysis was performed on patients initially eligible (development of moderate respiratory distress during hospitalization) but who gave consent to CPAP/NIV treatment only in a severe stage of respiratory distress.

The data obtained in this population (group-C) were compared to those of patients who had started CPAP/NIV at a 100 < P/F ≤ 150 (group-B) and to those obtained from all patients with moderate ARDS (group-A + group-B). Baseline characteristics and outcomes of these populations are summarized in Tables 1 and 2.

The three groups (A + B vs. B vs. C) are comparable for baseline characteristics such as age, gender, CCI and comorbidities (Table 1). Prevalence of chronic obstructive pulmonary disease (COPD) appears significantly higher in moderate ARDS group (group A + B and group B) than in the severe one (group-C). Furthermore, no statistically significant difference emerged at baseline neither for blood tests and gas analysis, nor for radiological severity scores. All group C patients were treated with HFNC before they consent to CPAP/NIV treatment.

All patients were treated with LMWH and steroids; tocilizumab were given in 13.4%, 14.7% and 12.9% in group A + B, B and C respectively (Table 2). A higher statistically significant percentage of remdesivir-treated patients (33% in group A + B, 29.4% in group-B and 9.7% in group-C) was seen in groups with progressively less severe ARDS, consistent with the drug’s datasheet (not indicated in patients on HFNC or mechanical ventilation) [27].

Table 2 shows the outcomes of these populations. In the comparison between group B and C was observed a growing trend for in-hospital mortality, even though not statistically significant, for patients undergoing NRS treatment in severe ARDS (13.2% in group-B vs. 29% in group-C, p = 0.059) (Table 2). Furthermore, patients undergoing NRS treatment in the severe ARDS phase needed more continuous rather intermittent CPAP/NIV (22.1% and 54.8% in group-B and C, respectively—p = 0.002) and PS through conversion from CPAP to NIV (19.1% and 48.4% in group-B and C, respectively—p = 0.002). No statistically significant difference emerged, instead, for pressures (PEEP/PS) required, overall ventilation and hospitalization length or days between the start of NRS and OTI or weaning.

The comparison of combined data of patients undergone NRS in moderate (100 < PF ≤ 200) and severe stage of ARDS (P/F ≤ 100) showed that starting NRS treatment in the severe phase of ARDS is associated to a significant increase in in-hospital mortality as compared to moderate stage (29.0 vs. 13.4%, p = 0.044). On the other hand, the two populations do not disclose any statistically significant difference in OTI rate (12.9% severe group, 10.3% moderate group, p = 0.69). Indeed, severe stage group also shows a statistically significant increase in the median hospitalization length (15 vs. 14 days in moderate group, p = 0.038) and need for continuous ventilation (54.8 vs. 22,7% in moderate group, p = 0.0007) or PS (48.4 vs. 18.6% in moderate group, p = 0.003).

Figure 3 shows overall survival and OTI rate curves of patients underwent CPAP/NIV in these populations. Patients who started NRS in ARDS-moderate stage (Fig. 3c) show a significantly higher survival than patients undergone NRS in a severe stage (p = 0.038). No statistically significant difference emerged in OTI rate (Fig. 3d, p=0.667).

### Predictors of CPAP/NIV failure

Potential predictors of CPAP/NIV failure are shown in Table 3. The composite outcome of death and need for OTI is defined as "failure". On univariate analysis, age (HR 1.152; CI 1.084–1.2224, p < 0.001), CCI (HR 1.514; CI 1.274–1.798, p < 0.001), need for conversion to PSV (HR 0.135; CI 0.048–0.3777, p < 0.001) and continuous rather than intermittent ventilation (HR 0.046; CI 0.006–0.355, p = 0.003) found to be significantly associated with CPAP/NIV failure. At multivariate analysis, independent predictors of CPAP/NIV failure were instead advanced age (HR 1.147; CI 1.076–1.2222, p < 0.001) and need for continuous ventilation (HR 0.031; CI 0.003–0.328, p = 0.004).

**Table 3:** Assessment of potential predictors of the composite of mortality and OTI in the overall population

Variables	Univariate analysis			Multivariate analysis		
	HR	95% CI	p	HR	95% CI	p
Age	1.152	1.084–1.224	** < 0.001**	1.147	1.076–1.222	** < 0.001**
Sex	0.811	0.319–2.059	0.659			
CCI	1.514	1.274–1.798	** < 0.001**	0.907	0.610–1.351	0.632
Duration of disease pre-admission	0.908	0.804–1.024	0.116			
D-dimer	1.000	1.000–1.000	0.278			
LUS Score	0.939	0.829–1.064	0.326			
CT Score	1.400	0.801–2.446	0.238			
Conversion to NIV	0.135	0.048–0.377	** < 0.001**	0.581	0.153–2.203	0.424
Days of ventilation	0.954	0.846–1.075	0.438			
Continuous ventilation	0.046	0.006–0.355	**0.003**	0.031	0.003–0.328	**0.004**

Statistically significant values in bold

*ARDS* Acute Respiratory Distress Syndrome, *CCI* Charlson Comorbidity Index, *CI* Confidence Interval, *CT* Computed Tomography, *HR* Hazard Ratio, *LUS* Lung Ultrasound, *NIV* Non Invasive Ventilation, *OTI* orotracheal intubation, *P/F* PaO_2_/FiO_2_ rate

## Discussion

The efficacy of CPAP or NIV in the management of patients with SARS-CoV-2-related ARDS have not been established yet, since the available data are controversial. While some studies reported that patients undergoing CPAP, and then IMV, showed high mortality rates [3, 28], others have demonstrated a positive impact on survival [5–10, 29–33]. Our data show a survival rate of 87% (84/97) among patients undergone CPAP/NIV with moderate ARDS and of 83% (106/128) in all populations. Results from our study agree with those recently reported by Brusasco et al. [5], who showed a 94% survival rate in patients with moderate to severe ARDS undergone CPAP.

One of the most important concerns was that NRS could delay IMV those affecting survival rate. Recently, Menzella et al. [8] highlighted that NRS failure resulting in OTI does not lead to excess of mortality as compared to those continuing NIV even with evidence of failure. Perkins et al. [10] confirmed that a trial with CPAP significantly reduces mortality and OTI rate compared to conventional oxygen therapy. In a meta-analysis, the mortality rate among patients undergoing IMV after a trial of NIV seems comparable to that found among patients undergoing primary OTI (48.9% vs. 42.5%, p = 0.08) [9]. Therefore, the meta-analysis demonstrated that risks associated with delayed IMV in SARS-CoV-2-related ARDS are negligible and a CPAP/NIV trial would lead to a reduction of OTI rate. Moreover, mortality rate in patients undergoing IMV seems significantly high, up to 97% during first pandemic waves [4, 9, 11–16]. Thus, early use of IMV should be avoided since it could potentially worsen patient’s outcomes and lead to unjustified ICU overload. In this regard, recent Italian guidelines support the use of CPAP/NIV in patients with SARS-CoV-2-related hARF when standard oxygen support seems no longer sufficient, though not requiring immediate OTI [18].

Given that CPAP/NIV is probably effective in treating COVID-19-related ARDS, no data are available about the best timing to start non-invasive treatment that would maximize results and minimize side effects and waste of resources (equipment/dedicated personnel). The Italian guidelines [18] underline the need to define standardized criteria for both initiation and use of CPAP/NIV in COVID-19 patients. Although complications (e.g., pulmonary embolism) may occur in the clinical course of COVID-19 regardless of stage of disease, in the natural history of SARS-CoV-2-related ARDS, respiratory distress severity is usually progressive, but the rate of damage progression is extremely variable [34]. Thus, the most appropriate timing to start treatment during SARS-CoV-2 infection cannot be defined by the days since the onset of the disease, rather by the severity of the clinical picture. In this context, the P/F ratio is the parameter that best expresses the degree of severity of respiratory distress and therefore can be crucial in defining the timing of intervention [20]. All trials evaluating the efficacy of NRS in this setting use extremely heterogeneous and arbitrary cut-offs (P/F ≤ 150, ≤ 200, ≤ 250, ≤ 300) [1, 5, 6, 8, 35], without well-specified criteria to start NRS [3, 9, 29, 31, 32, 35–37], affecting results and proposed algorithms.

To our knowledge, our study is the first to evaluate the impact of initiating CPAP/NIV at different levels of respiratory distress severity. In our study, there were no significant differences in outcomes (mortality, OTI rate, hospital days) among patients with moderate respiratory distress who started CPAP/NIV when the P/F was between 151 and 200 compared to patients initiated non-invasive treatment when the P/F was between 101 and 150. Data indicate that starting NRS treatment in the earlier stage of moderate ARDS (group-A) does not significantly reduce in-hospital mortality rates compared to starting treatment in the later stage (group-B, 13.8 vs. 13.2%, p = 1), as well as the need for PS or continuous ventilation, OTI rate and hospitalization length (Table 2). Conversely, this would potentially lead to an increased waste of resources (equipment/dedicated personnel) and a greater and unjustified risk of ventilator-associated lung injury (VILI, potential but not found in our series) or deep vein thrombosis (when helmet is chosen as interface) [24, 38].

Patients who initiated treatment at 100 < P/F ≤ 150 show a clear downward trend in in-hospital mortality compared to patients with a P/F ≤ 100 at NRS starting (13.2% in group-B vs. 29% in group-C, p = 0.059). These data appears to be of clinical relevance even though not statistically significant due to the low sample size. From the analysis of combined data of patients undergone NRS in the moderate ARDS phase (group A + B) or severe (group C) emerged that patients who started the treatment in a severe stage show a statistically significant increase in the mortality rate (29%) comparing to patients treated with CPAP/NIV in a moderate stage (13.4%, p = 0.044, Table 2). Therefore, data suggest that delaying CPAP/NIV in a severe stage of ARDS leads to an unjustified increase in in-hospital and overall mortality rate (Fig. 3c).

On the other hand, our data do not show a significant impact of NRS timing on OTI rate, likely in relation to the low number of cases (Table 2). Indeed, trials focused on the absolute OTI risk during SARS-CoV-2-related hARF showed that CPAP is associated to a reduction in the need for IMV in 35–50% of cases [6, 8, 10, 35]. Oranger et al. showed a higher reduction in the need for OTI (75%) following the use of CPAP [7]. In a recent Italian trial [1], Grieco et al. showed that continuous NIV leads to a reduction in OTI rate compared to HFNC (30% vs. 51%, p = 0.03).

Our findings also underline that the initiation of CPAP/NIV in the moderate ARDS phase significantly reduces the median hospitalization length compared to starting treatment in the severe stage (14 days, group A + B vs. 15 days, group C, respectively; p = 0.038, Table 2) and could help in optimizing the availability of beds, a crucial issue during pandemic. Moreover, starting CPAP/NIV in a severe stage leads to a significantly greater need for PS (due to CPAP failure) and continuous ventilation (> 18 h/day), potentially increasing the risk of VILI and need for total parenteral nutrition, as well as the risk of sepsis and electrolytic disorders. In this regard, the two populations undergone NRS in a moderate stage at a P/F level above or below 150 show the same outcomes, underlying that early CPAP/NIV (P/F > 150) does not positively affect the need for PS or continuous ventilation, as mortality, OTI rate and hospitalization length.

**Fig. 4:**
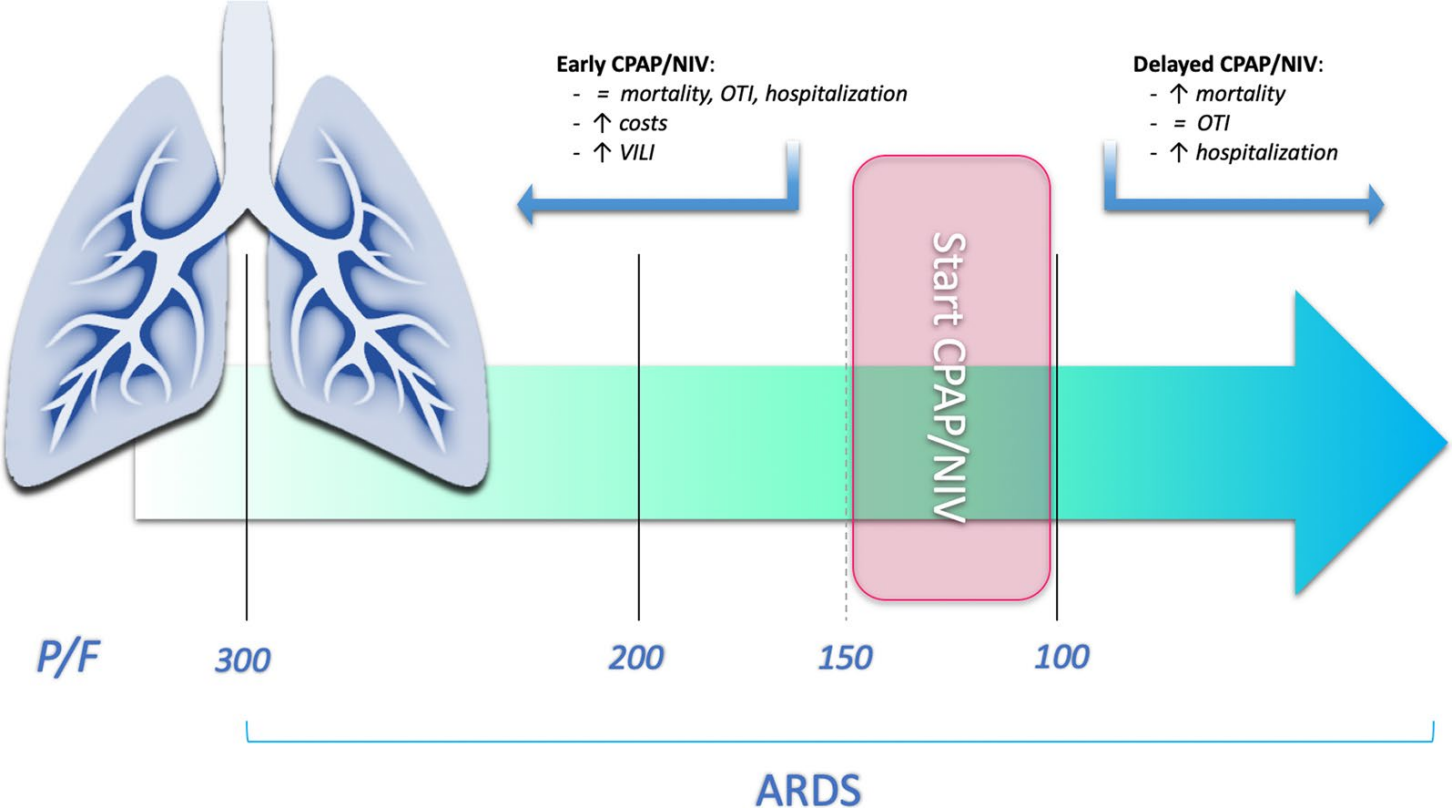
Proposed timing for start CPAP/NIV to maximize results to be evaluated in future powered studies. *ARDS* Acute Respiratory Distress Syndrome, *CPAP* Continuous Positive Airway Pressure, *NIV* Non-Invasive Ventilation, *OTI* oro-tracheal intubation, *P/F* PaO2/FiO2 rate; *VILI* Ventilator Induced Lung Injury

Overall, our findings, consistent with those from the most recent trials [1, 5, 8, 10], suggest that an appropriate use of NRS could reduce in-hospital mortality in SARS-CoV-2 related ARDS [39]. To our knowledge, our study is the first to evaluate the most appropriate timing to initiate non-invasive treatment comparing the efficacy of starting NRS in patients at different severity levels of respiratory distress. The integration of all performed analyzes suggests that starting CPAP/NIV in a moderate ARDS stage (100 < P/F ≤ 200) would allow to optimize outcomes. Although data obtained do not allow definitive conclusions due to small sample size, mortality trend suggests that the best window to start treatment with CPAP/NIV could be probably that of P/F between 100 and 150. Starting NRS treatment in this range could be cost-effective, minimizing on one hand in-hospital mortality rate and hospitalization length (higher for delayed treatments at P/F ≤ 100) and, on the other, the waste of resources in terms of equipment and dedicated personnel, as well as potential VILI (due to similar outcomes for treatment started with a P/F above or below 150). Moreover, advanced age and need for non-invasive continuous ventilation seems to be predictors of CPAP/NIV failure. Assuming the difference in mortality rates between patients underwent NRS at a P/F level between 150 and 100 compared to those underwent treatment in a severe phase of ARDS clinically relevant, although not showing statistical significance, we suggest a possible flow chart for starting NRS treatment in patients with SARS-CoV-2-related ARDS that needs to be validated in future powered studies (Fig. 4).

The main limitation of our research is due to its exploratory study design which does not reach uniformity in the sample size as well as an adequate power because of the small sample size. However, it should be emphasized that no significant differences in baseline parameters were found between our subpopulations. No significant differences emerged for the main risk factors known for adverse outcomes (age and CCI) and comorbidities. For these reasons, we are persuaded that the data can be considered with sufficient confidence.

As reported in the study methods, a sequential ventilatory support has been used (from conventional oxygen to CPAP, to NIV, to OTI). However, if the role of CPAP in the management of ARDS is being defined, to date no strong evidences about the role of NIV are available. As not yet validated, NIV treatment after CPAP failure could be not appropriate and could have affected the results by delaying OTI. For the same reasons, the ventilation pressures used (PEEP, PS) could be arbitrary and, potentially, have influenced the results. A further limitation is that, due to the absence of reference data, an arbitrary P/F cut-off of 150 was chosen in the assessment of the timing to start CPAP/NIV in patients with moderate ARDS. Therefore, it cannot be considered an absolute reference value. However, our data indicate that early treatment with CPAP/NIV in moderate ARDS stage has no significant clinical benefit and exposes the patients to the risks related to ventilation. Thus, for the mentioned reasons, our data must be considered preliminary and confirmed in future studies with adequate power.

Caution is also required about the use of P/F ratio, which seems fundamental in stratification of ARDS severity and in the choice of most appropriate therapies, but it does not represent the only factor to be considered in the evaluation of the outcomes. Hence, a tailored treatment appears mandatory to define the most suitable therapeutic approach in each case. Different pathogenetic mechanisms (e.g.: coagulopathy and pulmonary vascular thrombosis) and different phenotypes of interstitial pneumonia may in fact require diversified therapeutic approaches.

## Conclusions

In conclusion, data from this pilot study indicate that starting CPAP/NIV treatment in patients with SARS-CoV-2-related ARDS in moderate stage is associated to a significative reduction of in-hospital mortality and length of hospitalization compared to treatment started in severe stage. It seems likely that the distress severity stage to start CPAP/NIV able to maximize results ranges a P/F between 101 and 150. An earlier start does not lead to significant differences in mortality, OTI rate and hospitalization length, resulting only in waste of resources and potential VILI. Advanced age and the need for continuous ventilation emerged as independent predictors of CPAP/NIV failure. However, the data require confirmation from studies with adequate power.

## Data Availability

The datasets used and/or analysed during the current study are available from the corresponding author on reasonable request.

## Abbreviations

AIFA: Italian drugs agency
ARDS: Acute respiratory distress syndrome
CCI: Charlson comorbidity index
CI: Confidence interval
COPD: Chronic obstructive pulmonary disease
CPAP: Continuous positive airway pressure
CT: Computed tomography
DNI: Do not intubate
FFP: Filtering face-piece
FiO_2_: Delivered oxygen
GCS: Glasgow coma scale
hARF: Acute hypoxemic respiratory failure
HFNC: High flow nasal cannula
HR: Hazard ratio
HRCT: High-resolution chest tomography
ICUs: Intensive care units
IMV: Invasive mechanical ventilation
IQR: Interquartile range
LMWH: Low-molecular weight heparin
LUS: Lung ultrasound score
NIV: Non-invasive ventilation
NRS: Non-invasive respiratory support
OTI: Oro-tracheal intubation
PEEP: Positive end-expiratory pressure
P/F: PaO_2_/FiO_2_ rate
PS: Pressure support
PSV: Pressure support ventilation
RR: Respiratory rate
SD: Standard deviation
SpO_2_: Peripheral oxygen saturation
TVs: Tidal volumes
VILI: Ventilator-associated lung injury
